# Quantitative analysis of single-molecule force spectroscopy on folded chromatin fibers

**DOI:** 10.1093/nar/gkv215

**Published:** 2015-03-16

**Authors:** He Meng, Kurt Andresen, John van Noort

**Affiliations:** 1 Biological and Soft Matter Physics, Huygens-Kamerlingh Onnes Laboratory, Leiden University, Leiden, The Netherlands; 2 Department of Physics, Gettysburg College, Gettysburg, PA 17325, USA

## Abstract

Single-molecule techniques allow for picoNewton manipulation and nanometer accuracy measurements of single chromatin fibers. However, the complexity of the data, the heterogeneity of the composition of individual fibers and the relatively large fluctuations in extension of the fibers complicate a structural interpretation of such force-extension curves. Here we introduce a statistical mechanics model that quantitatively describes the extension of individual fibers in response to force on a per nucleosome basis. Four nucleosome conformations can be distinguished when pulling a chromatin fiber apart. A novel, transient conformation is introduced that coexists with single wrapped nucleosomes between 3 and 7 pN. Comparison of force-extension curves between single nucleosomes and chromatin fibers shows that embedding nucleosomes in a fiber stabilizes the nucleosome by 10 *k*_*B*_*T*. Chromatin fibers with 20- and 50-bp linker DNA follow a different unfolding pathway. These results have implications for accessibility of DNA in fully folded and partially unwrapped chromatin fibers and are vital for understanding force unfolding experiments on nucleosome arrays.

## INTRODUCTION

The condensation of meters of DNA into the nucleus of a eukaryotic cell requires dense packing of the DNA into a structure called chromatin. This organization of eukaryotic DNA has attracted increasing interest because it is now evident that epigenetic changes to chromatin provide the cell with a means to fine-tune the regulation of its genes (1). The physical mechanisms that are responsible for such epigenetic regulation clearly depend on the detailed structural arrangements of the molecules involved, but resolving the structure of chromatin at this scale has proven to be an enormous challenge.

The first level of DNA compaction, the nucleosome, is formed by wrapping 147 bp of DNA around a positively charged histone protein core (2,3). It is now well established that the nucleosome is a rather dynamic entity, allowing for spontaneous and force-induced DNA unwrapping (4,5), exchange of H2A-H2B histones (6) and thermal (7) and enzymatic repositioning (8,9). Several post-translational modifications have been shown to modulate the dynamics of these processes (10,11). Overall, single nucleosomes have been well characterized yielding a dynamic structure in which DNA can transiently unwrap from the histone core.

The next level of organization is much more elusive. Despite great insights into the structure of nucleosome arrays from crystallography (12), electron microscopy (EM) (13–15) and sedimentation analysis (16,17), our understanding of the folding of an array of nucleosomes into a condensed fiber is limited(18,19). Part of the difficulty in studying the structure of chromatin fibers is the heterogeneity of the fiber's composition. The use of tandem arrays of the synthetic Widom 601 DNA nucleosome positioning sequence (20) for making well-defined nucleosomal arrays has greatly aided the study of chromatin folding (13), but still there is no consensus on the structure of chromatin. In fact, these regular arrays may not be representative for the situation *in vivo* (21), where nucleosomes can be distributed along the DNA with irregular spacings, though regular nucleosome spacings have been reported downstream of transcriptions start sites (22). The consequences of differences in nucleosome spacing for chromatin folding can be large, as small changes in linker length can have a large impact on the overall structure of a chromatin fiber (19,23–25). Rather than looking for regular higher order structures, it may therefore be more illuminating to characterize the interactions between nucleosomes that define the folding of nucleosomal arrays into condensed chromatin fibers.

Single-molecule force spectroscopy is a powerful tool for probing molecular interactions. Pulling experiments on single nucleosomes reconstituted on a long DNA fragment containing a single 601 element revealed a detailed picture of force-induced DNA unwrapping (5). Two transitions were described, one at ∼3 pN, corresponding to the unwrapping of about one turn of DNA, followed by a higher force (∼8–9 pN) transition, representing the unwrapping of the remaining DNA. Such three-state behavior has since been confirmed by others (26–28). The low-force unfolding transition is reversible. Constant force measurements allowed for quantification of the free energy and rate constants of wrapping and unwrapping. The second transition is only reversible when the force is reduced to several pN. Theoretical modeling has indicated that the bending of linker DNA plays an important role in defining the structures of these meta-stable conformations (29). The stability of a nucleosome under tension is therefore related to the DNA handles that are used to pull on it.

Nucleosome arrays have also been subject to manipulation with optical and magnetic tweezers (MTs). Early work on nucleosome arrays largely focused on the high-force unwrapping transition(4,30). The equivalent of ∼72 bp is released in a step-wise irreversible fashion at 10–20 pN. At such forces the increased distance between the nucleosomes, due to stretching and unwrapping, is large enough to exclude interactions between nucleosomes. Only a few studies have focused on the low-force regime (31,32), where a level of condensation is found of 2–3.6 nm/nucleosome, approaching an extension of 1.2 nm/nucleosome as observed by EM (13). Force-extension curves in this low-force regime feature a transition to a large extension at ∼3 pN as well. It is therefore non-trivial to distinguish DNA unwrapping, as observed in mononucleosomes, from the possible disruption of direct nucleosome–nucleosome interactions in folded chromatin fibers.

These single-molecule force spectroscopy data, as well as other structural studies, have led to a wealth of theoretical descriptions of the structure and mechanical properties of chromatin fibers. Full atom simulations (25) yield the most detailed structures, resulting from geometric constraints of the nucleosome positions and realistic mechanical properties of the linker DNA. Coarse-grained numerical models (16,33–36) allow for a wider range of structural parameters, including variations in position and post-translational modifications of the histone tails. More analytical approaches (37–40) emphasize the mechanical properties of the linker DNA, while ignoring the detailed structure and composition of the histone proteins. While these works have set physical boundaries for the parameters that describe chromatin folding, most models are not detailed enough or use too many parameters to directly retrieve physical parameters from the experimental force spectroscopy data.

Here we aim to disentangle unfolding transitions in chromatin fibers, using new experimental data as well as a novel quantitative model for all aspects of a force-induced unwrapping of a chromatin fiber. The model uses statistical mechanics to describe transitions between four conformations of each nucleosome in the fiber. With this statistical mechanics model we quantitatively compare pulling traces of mononucleosomes with those of fully folded fibers. Despite using arrays of Widom 601 positioning elements and careful titration of the reconstitution dialysis (13), we find it necessary to include some heterogeneity of the chromatin fibers in terms of nucleosome composition. When these heterogeneities are accounted for, we are able to determine consistent values for DNA unwrapping free energies and extensions of each nucleosome conformation. A novel intermediate conformation is exposed, existing between 2.5 and 7 pN. Moreover, the qualitative difference in rupture behavior between chromatin fibers with 197-bp nucleosome repeat lengths (NRLs) and 167-bp NRL may indicate a different folding topology. Finally, by comparing the thermodynamical parameters of a mononucleosome with those of nucleosomes in a folded chromatin fiber we unequivocally resolve the magnitude of stabilization of nucleosomes embedded in a folded fiber.

## MATERIALS AND METHODS

### Chromatin reconstitution

A DNA substrate based on pUC18 (Novagen) with inserts containing 15 times 197-bp and 30 times 167-bp repeats of the Widom 601 nucleosome positioning sequence was used for reconstitution of chromatin fibers. After digestion with BsaI and BseYI enzyme, single-stranded ends were filled with a dUTP-digoxigenin at the BsaI and a dUTP-biotin at the BseYI end by Klenow reaction. The linear DNA fragment was mixed with 147-bp competitor DNA and histone octamers purified from chicken erythrocytes, and reconstituted into chromatin fibers using salt dialysis following (13).

### Sample preparation

A clean cover slip was coated with 1% polystyrene-toluene or 0.1% nitrocellulose in amylacetate solution and mounted on a poly-di-methysiloxane (PDMS, Dow Corning) flow cell containing a 10 × 40 × 0.4-mm^3^ flow channel. The flow cell was incubated with 1-μg/ml anti-digoxigenin for 2 h and 2% bovine serum albumin (w/v) solution over night. Twenty-nanogram/milliliter fibers in 10-mM Hepes pH 7.6, 100-mM KAc, 2-mM MgAc_2_ and 10-mM NaN_3_ were flushed into the flow cell and incubated for 10 min, followed by flushing in 2.8- μm streptavidin-coated superparamagnetic microspheres (M270, Invitrogen) in the same buffer. Loose beads were flushed out after another 10 min of incubation.

### Magnetic tweezers

The home-build MTs have been described by Kruithof *et al*. (41). During an experiment, a single chromatin fiber was tethered between the end of a superparamagnetic bead and the surface of a microscope coverslip. The force was varied by moving the pair of magnets at 0.1 mm/s. The extension of the DNA was measured in real time at a frame rate of 60 Hz with a CCD camera (Pulnix TM-6710CL).

### Data analysis

Data analysis and curve fitting was done using a custom software written in LabVIEW. The offset of each force-extension curve was adjusted by aligning the extension at high force, after the last rupture event, with a Worm Like Chain (WLC) using the known contour length, a persistence length of 50 nm and a stretch modulus of 1200 pN. This procedure circumvents errors due to off-center attachment (42,43) and the roughness of the bead and surface. In some cases a linear drift was subtracted to enforce overlap from successive pulling experiments. This drift correction was validated by the (partial) overlap of pull and release curves. All data are presented and analyzed without further filtering or averaging.

Rupture events at high force were automatically detected with a *t*-test step finding algorithm, using a 10-point window (44). At forces larger than the first rupture event the fitted extension was assigned to the extension of the state that matched the experimental data point best. To eliminate erroneous assignments due to the relatively large amplitude of thermal fluctuations in the extension this part of the fitted curve was filtered using a 10-point median filter. At forces below the first rupture event the data were fitted to Equation (8) using a Levenberg–Marquardt algorithm. Instabilities due to the discrete nature of the number of nucleosomes were circumvented by linear interpolation of these parameters. Generally, the fit results yielded numbers of nucleosomes that were within 0.1 of an integer number. Data points acquired at forces below 0.5 pN were not included in the fit to exclude artifacts due to bead–surface interactions.

## RESULTS

### Nucleosomes unfold differently in chromatin fibers as compared to mononucleosomes

**Figure 1.**
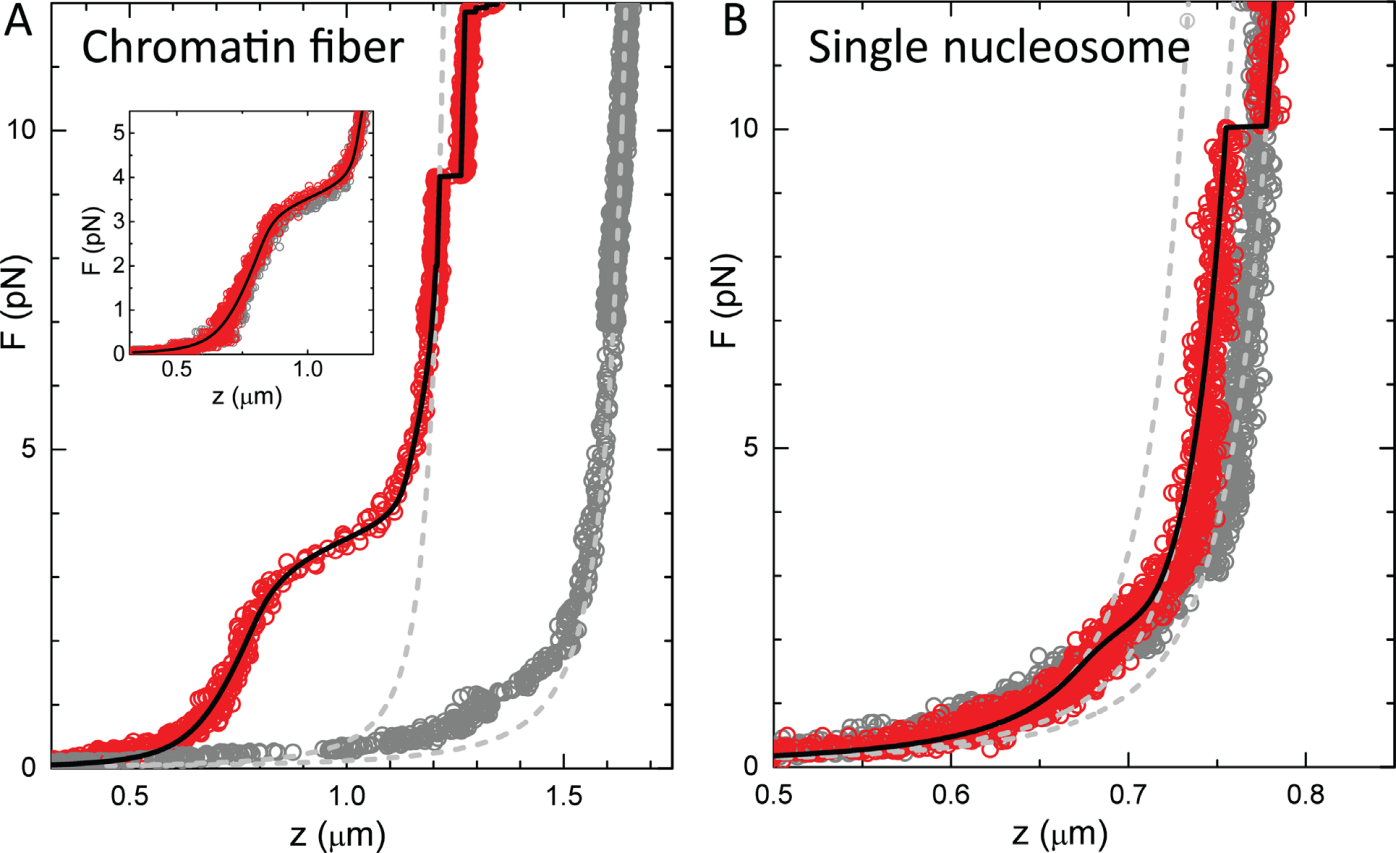
Comparison between force-extension curves of (**A**) a chromatin fiber and (**B**) a mononucleosome. Dark circles represent the pulling trace, light gray circles represent the release trace. All force-extension measurements are reversible, but a significant hysteresis is observed when the the force exceeds 6 pN. The inset in (A) shows a force-extension experiment in which the force was limited to 6 pN; no hysteresis is observed. Light gray dashed lines represent WLC descriptions of the bare DNA and the state in which all nucleosomes are in the extended conformation (see Figure 2). A third dashed line in (B) represents a WLC with a contour length 147 bp shorter than the bare DNA. Black lines are fits to Equation (8) yielding for (A) *n*_fiber_ = 13, *n*_unfolded_ = 4, *k* = 0.28 pN/nm, $z$_ext_ = 4.6 nm, Δ*G*_1_ = 20.6 *k*_*B*_*T* and Δ*G*_2_ = 5.5 *k*_*B*_*T*. For (B): $z$_ext_ = 6.5 nm, Δ*G*_1_ = 8.8 *k*_*B*_*T* and Δ*G*_2_ = 3.5 *k*_*B*_*T*.

To capture all aspects of chromatin folding we measure and analyze here the force-extension relation of single chromatin fibers from small, sub-pN forces up to several tens of pN. Figure 1A shows a force-extension curve of a chromatin fiber reconstituted with a tandem array of 15 repeats of a 197-bp Widom 601 nucleosome positioning sequence. A slow increase in extension is observed between 0.5 and 3 pN, followed by an extension of several hundred nanometers as force increases and, starting at about 9 pN, multiple stepwise unfolding events. These features have been described before as stretching of the chromatin fiber (32), rupture of roughly one turn of DNA from each of the nucleosomes (4) and at last the rupture of the second wrap of DNA from the histone core.

Whereas the stepwise unwrapping events at high force can unequivocally be attributed to the rupture of individual nucleosomes, the low-force events are more difficult to interpret. It has been suggested that this characteristic force-extension relation at forces below 10 pN can be understood without nucleosome–nucleosome interactions and represents the gradual unwrapping of the outer turn of DNA from the nucleosomes (35). Indeed, the force-extension trace of a single nucleosome under identical conditions, shown in Figure 1B, has remarkably similar characteristics, featuring three stages of unwrapping in the same force regimes as reported before (5,40,45). Because there are no neighboring nucleosomes in this case, all events should be attributed to the rupture of histone–DNA contacts. However, closer inspection shows that the first force plateau is slightly lower for the mononucleosome, 2.5 pN, than for the chromatin fiber, 3.5 pN, suggesting additional nucleosome–nucleosome interactions that stabilize the nucleosome in a folded fiber.

Another difference between mononucleosomes and chromatin fibers is that the latter show a rather large variation in the force-extension curve at low force. Despite careful titration of the histone–DNA stoichiometry and selection of the best batch using native gel electrophoresis (13), we generally observe significant variations in the low-force regime. Previously, we circumvented this problem by selecting only the most condensed chromatin fibers (32), assuming that those would be fully reconstituted with nucleosomes. However, chromatin fibers can be unstable under the highly diluted conditions that are typically used for single-molecule force spectroscopy. Claudet *et al*. pointed out that H2A–H2B dimers can readily dissociate, leaving (H3–H4)_2_ tetrasomes on the DNA (46). Despite the dissociation of dimers, the characteristic stepwise rupture events at 7–20 pN remain, showing that their occurrence cannot be used as an indication for the presence of a full nucleosome, but rather reflect the number of tetramers in a particular nucleosomal array. The ability to resolve this heterogeneity between chromatin fibers is one of the unique features of single-molecule techniques, though the occurrence of such variations in composition complicates a quantitative interpretation of force-extension relations of chromatin fibers in terms of structure and interaction energies.

**Figure 2.**
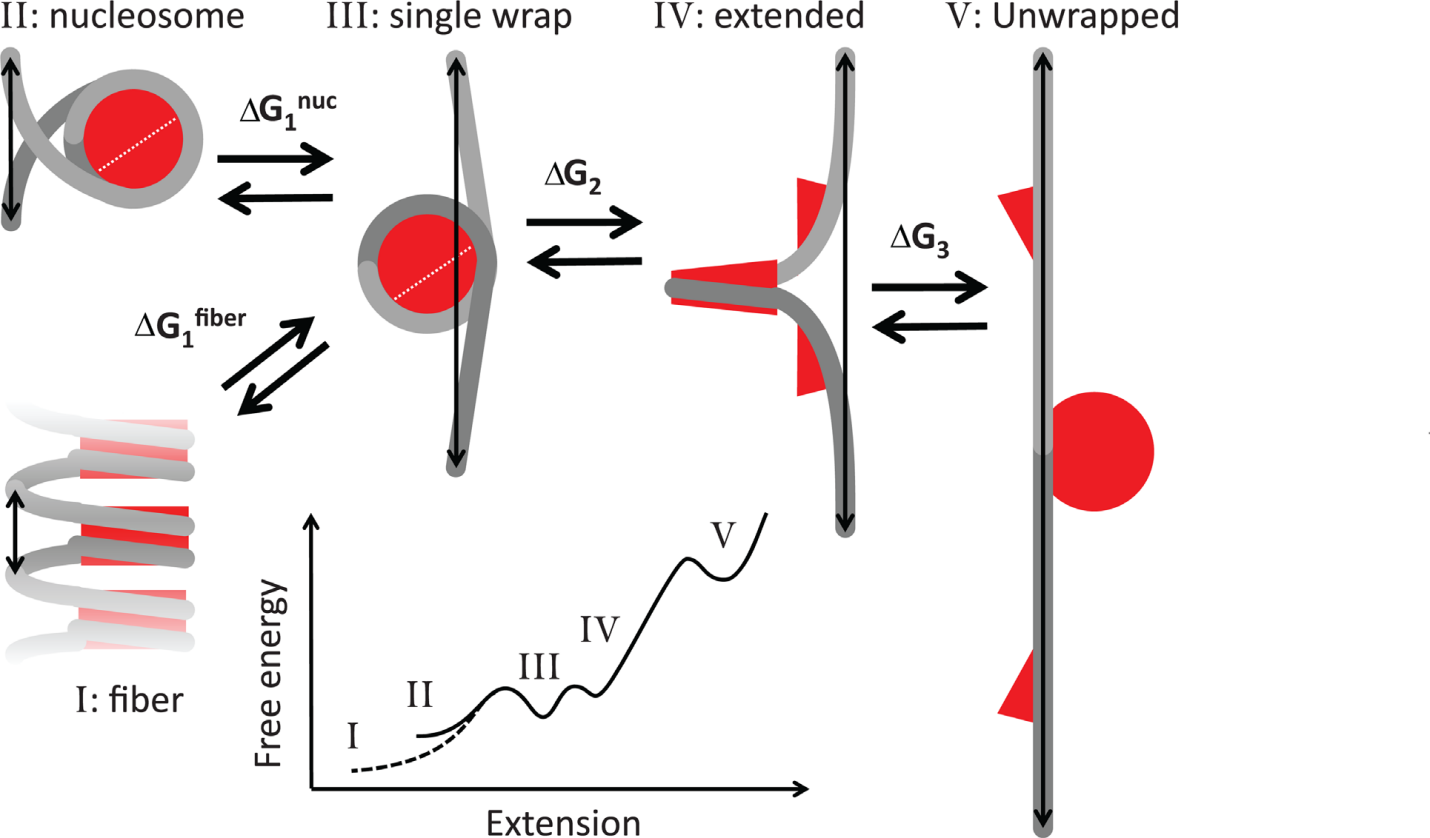
Schematic representation of the transitions between all metastable conformations of the nucleosomes. The double-headed arrows depict the extension per nucleosome for each conformation. As force increases, a nucleosome unwraps part of its DNA until a single full turn of DNA remains wrapped around the histone core. The next conformation is slightly extended, which may be due to further unwrapping of the DNA, conformational changes within the nucleosome and/or deformation of the linker DNA. We propose the extended conformation may involve dissociation of H2A/H2B dimers from histone core (see the Discussion section). In the last conformation all histone proteins remain attached to the DNA, but the DNA can stretch fully. When a nucleosome is embedded in a chromatin fiber and interactions between nucleosomes fold the fiber into a dense structure, the extension per nucleosome is further reduced, tentatively depicted as a stack of nucleosomes in the bottom left. After the first transition, involving a change in free energy of Δ*G*_1_, which may be different for a mono nucleosome and a nucleosome embedded in a fiber, all transitions will follow the same free energy landscape as schematically plotted in the inset.

In the next section we will set up a statistical mechanics framework that includes such heterogeneity. The thermodynamics is based on a free energy landscape for fiber unfolding that exhibits several metastable conformations, characterized by the roughness of the free energy landscape, as shown in Figure 2. The structures of the individual nucleosome conformations are tentatively depicted above the free energy diagram. Since the distance between the nucleosomes exceeds the length of the histone tails after the first transition (which could provide direct contact between nucleosomes), the Debye length and the deflection length of the DNA at these forces(47), each nucleosome follows the same unfolding pathway, independent of the number of nucleosomes in the fiber. This makes it possible to directly compare the unfolding of individual nucleosomes and distinguish their extension from that of the DNA linking the nucleosomes. For the folded fiber one cannot neglect interactions between nucleosomes, be it through direct contacts, electrostatics and/or mechanical coupling through the linker DNA. For such nucleosomes, folded in a fiber, we adopt an empirical approach and assume harmonic stretching of the fiber that scales with the number of nucleosomes. Importantly, all structural transitions up to the last unwrapping event are fully reversible within the time scale of the experiments, allowing for an equilibrium treatment of the transitions in the chromatin fiber. By careful quantification of the free energy and extension of each of the nucleosome conformations we aim to separate possible nucleosome–nucleosome interactions from DNA unwrapping from the histone cores, as measured in single nucleosomes.

### A multistate, statistical mechanics model

We describe a chromatin fiber as *n*_tot_ nucleosomes, which can be in any of the four conformations; see Figure 2. Here we explicitly test the scenario that nucleosomes in a folded chromatin fiber have different mechanical properties than a string of mononucleosomes in a beads-on-a-string structure. In addition to such nucleosomes embedded in a fiber, partially unfolded nucleosomes comprising one turn of DNA, and fully unwrapped nucleosomes in which all histones are still bound to the stretched DNA, we introduce a new metastable conformation in between the last two conformations, based on quantification of our experimental data (see the next section).

In our experiments the DNA substrate includes ∼1 kb of DNA handles that facilitate manipulation of the fiber. These DNA handles do not contain strong nucleosome positioning sequences and would, ideally, not contain any nucleosomes. The total extension of the tether, $z$_tot_, increases with force, *f*, as both the chromatin fiber and the DNA handles stretch elastically. On top of this elastic stretching, the nucleosomes will change conformation as force increases the fraction of nucleosomes in unwrapped, more extended conformations.

The extension of a DNA molecule follows an extensible WLC model (48):

(1) \begin{equation*} z_{{\rm DNA}}(f,\, L)=L\left[1-\frac{1}{2}\sqrt{\frac{k_{B}T}{f\, A}}+\frac{f}{S}\right] \end{equation*}

with contour length *L*, persistence length *A*, stretching modulus *S* and thermal energy *k*_*B*_*T*, yielding a free energy

(2) \begin{eqnarray*} &&G_{{\rm DNA}}(f,\, L)= \nonumber \\ &&-\int _{0}^{f}z_{{\rm DNA}}(\tilde{f},\, L)d\tilde{f}=-L\left[f-\sqrt{\frac{f\, k_{B}T}{A}}+\frac{f^{2}}{2S}\right]. \end{eqnarray*}

When nucleosomes are reconstituted on the DNA, the contour length of the free DNA is reduced by the amount of DNA that is wrapped around the histone cores. In the case of a single nucleosome the contour length is reduced by 147 bp. The extension of a one-turn-wrapped nucleosome, including its linker DNA, follows Equation (1), where *L* equals the NRL minus 89 bp, the amount of DNA in a single full wrap around the histone core. For an array of one-turn-wrapped nucleosomes the same description applies because the extension of each of the nucleosomes exceeds the limits set by the histone tail length, the Debye screening length and the DNA deflection length under the used experimental conditions. The change in free energy for this conformation is comprised of a part for stretching the free DNA, following Equation (2), and a term for rupturing part of the wrapped DNA, $\Delta G_{1}^{{\rm nuc}}$. As shown below, the experimental data suggest an intermediate conformation between the one-turn-wrapped and the fully unwrapped nucleosome. We assign an additional extension $z$_ext_ and free energy Δ*G*_2_ − *f* $z$_ext_ to this conformation. In the most extended conformation, the fully unwrapped nucleosome, the extension resembles that of bare DNA and can be described by a WLC with a contour length that equals the NRL and an additional free energy Δ*G*_3_ that is required to rupture the remaining DNA from the histone core.

In the absence of interactions between nucleosomes the above four conformations would suffice to quantitatively describe the entire force-extension behavior of a chromatin fiber. When nucleosomes interact however, the linker DNA is also constrained, further reducing the extension per nucleosome. Structural coarse-grained models have indicated a reduced extension and a compliance that depends on the precise arrangements of the nucleosomes (49). An analytical model by Ben-Haim *et al*. (37) yielded a linear force-extension relation for folded chromatin fibers. Experimentally, we indeed observe a linear response for the force range in which the folded chromatin fiber is stable. Accordingly, we model a Hookean extension of a nucleosome in the folded fiber:

(3) \begin{equation*} z_{{\rm fiber}}(f)=f/k+z_{0}, \end{equation*}

with a stiffness of *k*. Note that this stiffness is expressed per nucleosome, as opposed to our previous report (32), in which we considered the stiffness of the entire fiber. In Figure 2, we tentatively depicted nucleosomes in a folded fiber as stacked, neighboring nucleosomes, but other conformations could yield a similar force-extension relation.

At the forces that we analyze here, i.e. *f*  > 0.5 pN, the fiber aligns with the force and rotational fluctuations of the fiber can be neglected. This extension is included by adding a contour length per nucleosome, $z$_0_, which corresponds to the nucleosome line density that can be obtained from EM micrographs (13,50). Importantly, this representation does not imply a structural model of the fiber, but it does suggest that the fiber is short and stiff enough that entropic contributions do not significantly reduce its extension, as opposed to a flexible polymer like DNA.

The free energy contribution of the fiber follows from integration of Equation (3):

(4) \begin{equation*} G_{{\rm fiber}}(f)=-\int _{0}^{f}z_{{\rm fiber}}(\tilde{f})\, d\tilde{f}=-f^{2}/2k-fz_{0}. \end{equation*}

The thermodynamic properties of each of the conformations *i*, as schematically drawn in Figure 2, are summarized in Table 1, in which all physical dependencies between the different conformations are explicitly captured in a minimal number of parameters.

**Table 1.** Structural and thermodynamic parameters per nucleosome for different conformations sketched in Figure 2

$\bf {i}$	*L*_*i*_(*bp*)	$\bf {\bf {z_{i}\,}(nm)}$	$\bf {G_{i}\,(k_{B}T)}$
Nucleosome	NRL − 147	$z$_DNA_(*f, L*_*i*_)	*G*_DNA_(*f, L*_*i*_)
Fiber	-	*f*/*k* + $z$_0_	−*f*^2^/2*k* − *fz*_0_
Single wrap	NRL − *L*_wrap_	$z$_DNA_(*f, L*_*i*_)	*G*_DNA_(*f, L*_*i*_)+▵*G*_1_
Extended	NRL − *L*_wrap_	$z$_DNA_(*f, L*_*i*_) + $z$_ext_	*G*_DNA_(*f, L*_*i*_)+▵*G*_1_+▵*G*_2_-*f*$z$_ext_
Unwrapped	NRL	$z$_DNA_(*f, L*_*i*_)	*G*_DNA_(*f, L*_*i*_)+▵*G*_1_+▵*G*_2_+▵*G*_3_

The extension and free energy of the entire tether, containing *n*_tot_ nucleosomes, can now simply be calculated by summing the contributions of each nucleosome conformation *i* and the DNA handles:

(5) \begin{eqnarray*} z_{{\rm tot}}(f) & = & \sum _{i}n_{i}z_{i}(f)+z_{{\rm DNA}}(f,\, L) \end{eqnarray*}

(6) \begin{eqnarray*} G_{{\rm tot}}(f) & = & \sum _{i}n_{i}G_{i}(f)+G_{{\rm DNA}}(f,\, L). \end{eqnarray*}

When the DNA contains multiple nucleosomes, the chromatin fiber can be in a large, but finite number of states that are defined by the distribution of nucleosome conformations along the tether, state = {*n*_fiber_, *n*_single wrap_, *n*_extended_, *n*_unwrapped_}. This number can be reduced significantly by grouping states that have an equal number of nucleosomes in each of the conformations, but are arranged in a different order. These states cannot be distinguished based on extension only and are taken care of by including a degeneracy factor, which is calculated from a binomial distribution between the pairs of conformations *i* and *j* in each state:

(7) \begin{equation*} D({\rm state})=\prod _{i<j}\left(\begin{array}{c}n_{i}+n_{j}\\ n_{i} \end{array}\right), \end{equation*}

similar to (51). The mean equilibrium extension of the fiber as a function of force can now be computed using standard statistical mechanics, summing over all states:

(8) \begin{equation*} <z_{{\rm tot}}(f)>=\frac{\sum _{{\rm states}\,}z_{{\rm tot}}(f)\, D({\rm state})\, e^{-G_{{\rm tot}}(f)/k_{B}T}}{\sum _{{\rm states}}\, D({\rm state})\, e^{-G_{{\rm tot}}(f)/k_{B}T}}. \end{equation*}

To calculate the force-extension curve, we numerically evaluated Equation (8) for each force.

For the last transition however, the large hysteresis demonstrates that the equilibrium condition is not met. In this force range we do not fit the force-extension relation to Equation (8), but rather minimize the difference between the measured extension and the extension of each of the states as defined by Equation (5). Thus we cannot obtain the difference in free energy Δ*G*_3_, but we can still asses the extension and distribution of all of the nucleosome conformations at any force.

### DNA unwrapping at high forces involves less than one full wrap

**Figure 3.**
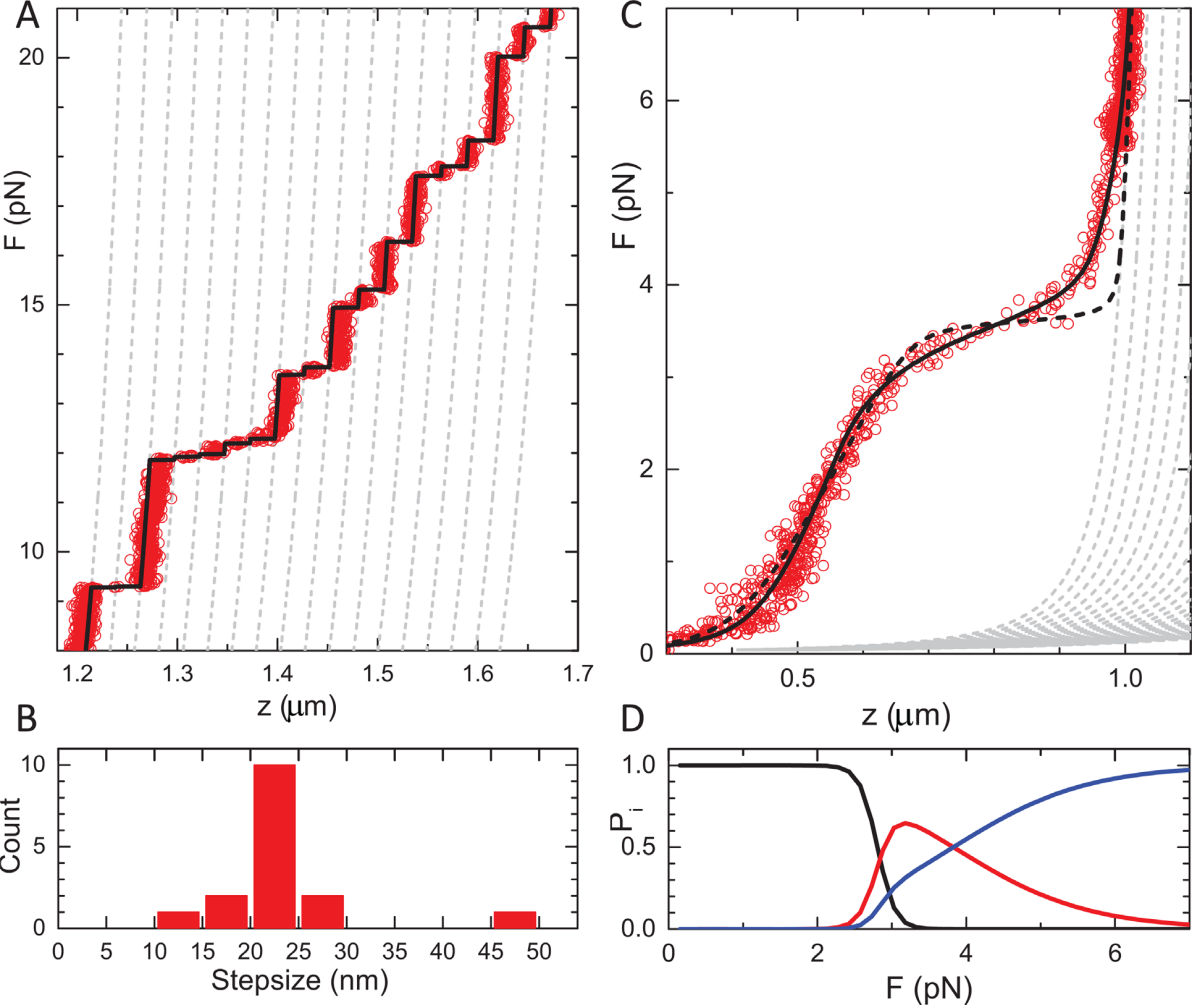
Detailed analysis of the unfolding of a single chromatin fiber. (**A**) A zoom in on the high-force region shows discrete steps in extension. Dashed gray lines represent the extensions of all states that are composed of extended and fully unwrapped nucleosomes. The fit match was obtained for $z$_ext_ = 4.6 nm. The black line shows the best match between individual data points and the various states of unwrapping. (**B**) Step size distribution of the data shown in (A) obtained from a 10-point window *t*-test analysis. (**C**) Unfolding of a 15*197 NRL chromatin fiber at low force. Below 7 pN the extension starts to deviate from a string of extended nucleosomes (gray dashed lines). A single transition (black dashed line) does not capture the force-extension data. The black line shows a fit to Equation (8), while constraining *L*_wrap_ = 89 bp and $z$_ext_ = 4.6 nm, yielding $\Delta G_{1}^{{\rm fiber}}$= 21.2±0.1 *k*_*B*_*T*, Δ*G*_2_= 4.3±0.1 *k*_*B*_*T*. (**D**) The corresponding probability for a nucleosome to be in a fiber (low force), a single wrap (intermediate force) or in the extended conformation (high force).

The discrete steps in extension at forces above 6 pN represent the sequential unwrapping of the last DNA from each nucleosome and have been studied abundantly with optical tweezers (4,27) and MTs (51). MTs act as a force clamp rather than a position clamp, resulting in a staircase-like force-extension curve instead of the typical saw-tooth pattern obtained with optical tweezers. Figure 3A shows a zoom in on these high-force transitions. The corresponding step size distribution is shown in Figure 3B. A step size of 22 ± 3 nm was found, in range with previous studies on various DNA substrates and under different buffer conditions.

It should be noted though that the reported step sizes vary significantly: 22 (5), 24 (46,51), 25 (27), 27 (4) and 30 nm (52). This high-force transition is generally interpreted as a conformational change from a nucleosome with one turn of wrapped DNA to the fully unwrapped nucleosome. Such a transition would involve the release of ∼89 bp of DNA, corresponding to ∼30 nm. We attribute the difference to a so-far-unresolved metastable conformation prior to full unwrapping, as schematically depicted in Figure 2. The extra extension of this conformation results in a large offset when multiple transitions occur in the same tether. For comparison we plotted the extension of each of the intermediate states that contain a mixture of this extended conformation and fully unwrapped nucleosomes in gray dashed lines. We obtained the best match between multiple independent experimental data sets and this intermediate state for $z$_ext_ = 4.6 nm. Indeed, only when this extended conformation of the nucleosomes is included, do the force-extension curves calculated with Equation (5) overlap with the experimental data and can each data point unequivocally be assigned to a specific state, as shown by the black line in Figure 3A. This analysis shows that the last transition involves less than a full wrap of DNA.

Because the high-force transitions are not in equilibrium it is not possible to extract the free energy Δ*G*_3_ that is associated with this transition. These high-force unfolding events are generally reversible however, when the force is decreased; see the inset in Figure 1A. This indicates that the histones do not dissociate from the DNA, though extended exposure to higher forces slowly reduces the number of observed transitions. Interestingly, the variation in step sizes is larger than the accuracy of the measurement (7 nm versus 2 nm) showing that not all nucleosomes behave exactly the same. In Figure 3A we observe for example a gradual extension between 10 and 12 pN, beyond what can be explained by a WLC and a specific number of one-turn-wrapped nucleosomes. This shift is made up for by a slightly smaller transition at 15 pN, after which the data accurately follow the theoretical curves again.

In the example trace shown in Figure 3A there are 17 clearly distinguishable steps, even though the chromatin fiber was reconstituted on 15 repeats of the 601 nucleosome positioning sequence. We frequently observed a mismatch between the number of high-force rupture events and the number of 601 repeats, demonstrating that the number of reconstituted nucleosomes is not strictly defined by the number of nucleosome positioning elements. The variation between individual fibers is small within a single reconstitution, and appears to depend on the precise histone/DNA ratio during reconstitution. Quantitative analysis of the high-force transitions allows for counting of the number of nucleosomes and/or tetrasomes that can both wrap at least one turn of DNA, in each fiber. Moreover, these transitions involve a conformation that is more extended than a nucleosome containing a single wrap.

### Fiber unfolding at low forces shows a novel unfolding intermediate

The force plateau at 3.5 pN represents the transitions from a folded fiber to a string of nucleosomes in an extended conformation, prior to the last unwrapping transition. A zoom in on this region for a fiber reconstituted on a 15*197 NRL DNA template is shown in Figure 3C. The experimental data only converge to the force-extension curve corresponding to the state with all nucleosomes in the extended conformation at 7 pN. Thus the unfolding of the fiber occurs in a rather large force region. Following our previous work, we fitted the extension of the folded chromatin fiber with a Hookean spring. The broad transition between the folded fiber and a string of extended nucleosomes cannot be captured in a single transition though, as shown by the black dashed line. We obtained a good fit by including two transitions, with the constraints *L*_wrap_ = 89 bp and $z$_ext_= 4.6 nm (as obtained from the discrete high-force rupture events), yielding $\Delta G_{1}^{{\rm fiber}}$= 21.2 ± 0.1 *k*_*B*_*T*, Δ*G*_2_= 4.3 ± 0.1 *k*_*B*_*T*. The necessity to include two transitions for an accurate description of the unfolding of a single chromatin fiber is a second indication that there is an additional metastable conformation of the nucleosome held under force.

Fitting the force-extension curve of a mononucleosome in this force regime, Figure 1B, results in an improved fit when the extended state is included, yielding $z$_ext_= 5.3 ± 0.5 nm, Δ*G*_2_= 5.0 ± 0.5 *k*_*B*_*T* and $\Delta G_{1}^{{\rm nuc}}$= 8.3 ± 0.2 *k*_*B*_*T*. The free energy for the first transition is very similar to previous reports (9.0 *k*_*B*_*T* by Mihardja *et al*. (5)) and can unequivocally be attributed to the unwrapping of DNA from the histone core. It therefore provides a good reference for comparison with chromatin fibers, in which nucleosome–nucleosome interactions may further stabilize DNA in the nucleosome. The fitted free energy of the first transition in unfolding the fiber is more than double of the value obtained for a single nucleosome, clearly demonstrating an extra stabilization of an embedded nucleosome by neighboring nucleosomes.

Using the parameters obtained above we plot in Figure 3D the probability of a nucleosome to be in each of the conformations that describe the fiber unfolding pathway. It is evident that multiple conformations coexist in a force region between 2 and 7 pN. This wide force range is due to the sequential order of events that only allow the second transition to occur when the first unfolding event has taken place. The smaller change in extension in this second step makes this transition less sensitive to force than the first unfolding transition.

One of the most striking features of these fits is that the unfolding of the chromatin fiber can be fully captured in four conformations, including the novel extended conformation. We could not resolve an intermediate conformation of a fully wrapped nucleosome without nucleosome–nucleosome interactions, often referred to as a beads-on-a-string structure. Imposing such a transition, as defined by the parameters obtained from the mononucleosome pulling experiment, further broadens the force plateau on the small extension side. The absence of such broadening, as we report here, may have important structural implications for the structure of a folded chromatin fiber.

### Variations between individual chromatin fibers result from heterogeneous fiber compositions

**Figure 4.**
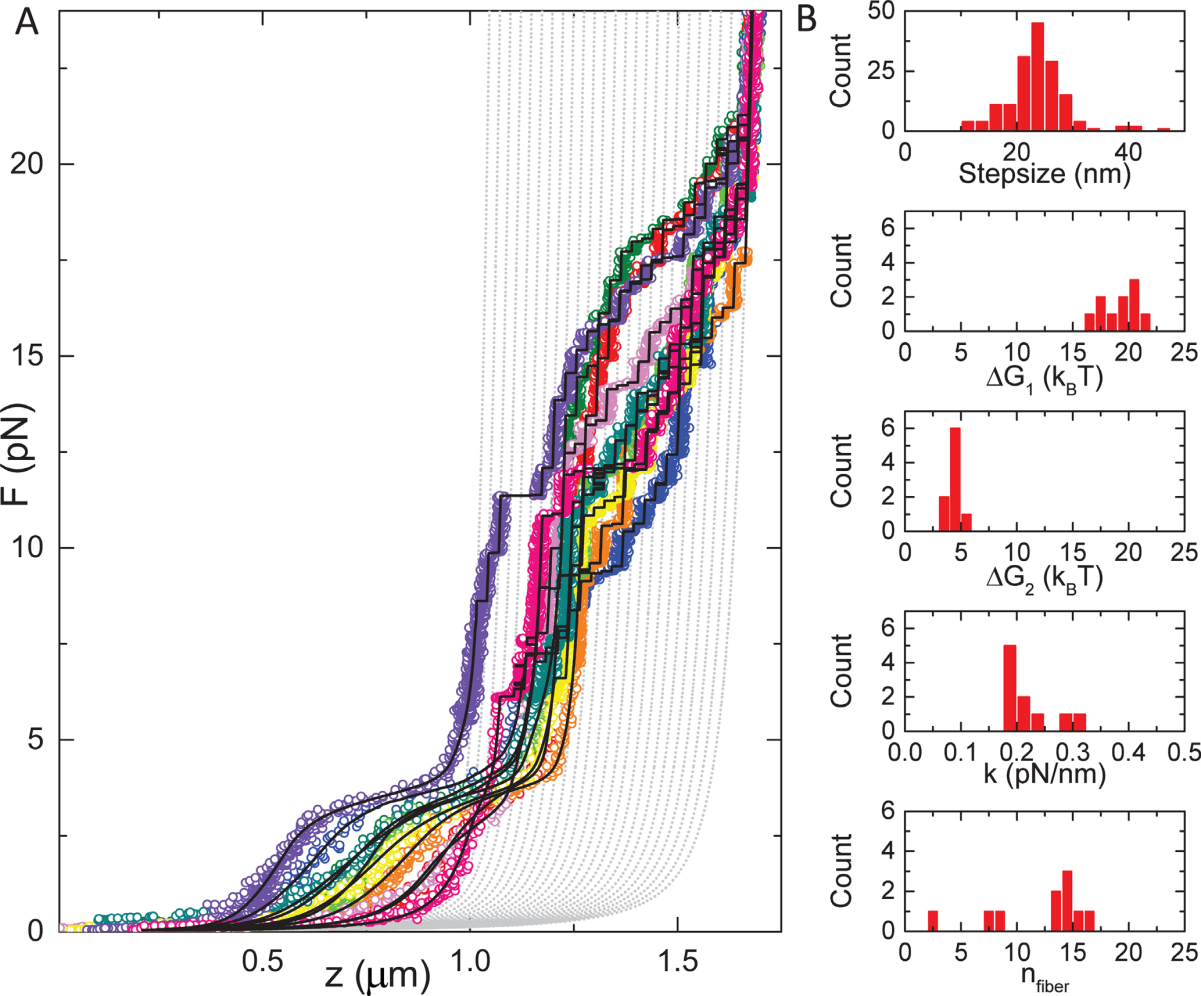
Different fibers show a large variation in condensation. (**A**) Ten chromatin fibers reconstituted on a 15*197 NRL DNA template. The high-force transitions align well with states that describe the last unfolding transition, plotted in gray dashed lines. All curves have a force plateau at 3 pN, but the size of the force plateau and the extension at lower forces varies significantly. Black lines represent fits to Equation (8). (**B**) Distribution of fit parameters obtained from (A). The stepsizes in the top histogram were determined independently using a *t*-test step finding algorithm. Except for the number of nucleosomes in the fiber, all parameters show a narrow distribution.

Whereas all chromatin fibers feature similar unfolding characteristics, we observed a rather large variation in the force-extension behavior between fibers. Figure 4 shows the force-extension curves of 10 different fibers. Because the last unfolding transition is not in thermodynamic equilibrium, the rupture forces for this transition are distributed stochastically. Nevertheless, all curves align well with the set of unfolding states that contain extended and fully unwrapped nucleosomes, indicated with the gray dashed lines. The first two transitions at forces below 7 pN on the other hand are fully reversible, resulting in overlapping pull and release curves (Figure 1A, inset). Despite the highly reproducible curves that we obtain from individual fibers, we observe large variations in extension between fibers in this low-force range. We attribute these differences to the variations in the composition of the fiber.

Oversaturation of the DNA substrate with nucleosomes, incomplete reconstitution and/or partial dissociation of nucleosomes after reconstitution may result in inhomogeneity of the fiber composition within a batch. Repetitive pulling cycles exceeding 5 pN, for example, show a gradual decrease of the condensation in the low-force regime (data not shown), which would be consistent with dissociation of several H2A–H2B dimers. Such a loss of H2A–H2B dimers would not only prohibit the formation of a fully wrapped nucleosome but also prevent nucleosome–nucleosome interactions that are thought to be mediated by interactions between the H4 tail and the acidic patch on the H2A–H2B dimer of a neighboring nucleosome (19). As a consequence, the number of rupture events at low force would be smaller than the number of rupture events detected at high force.

To deal with this heterogeneity we fitted the number of nucleosomes in the fiber, *n*_fiber_, independent of the number of nucleosomes that undergo the last transition, by introducing an additional parameter *n*_unfolded_, such that the total number of nucleosomal particles *n*_tot_ equals *n*_fiber_+*n*_unfolded_. The latter complexes, which we tentatively interpret to be tetrasomes, do not fold into a fiber or in a single wrap conformation, and only undergo the last unwrapping event. Importantly, we could not resolve separate populations in the last transition, suggesting that tetrasomes and nucleosomes indeed share the same last step in the unwrapping pathway. With this addition, all experimental curves gave good fits to the model and yielded a narrow distribution of fit parameters, as shown in Figure 4B and Table 2. The fitted number of nucleosomes in the fiber gave a much better correlation with the number of nucleosome positioning elements in the DNA substrate, but we still do not observe a perfect match. A quantitative interpretation of the force-extension data therefore requires analysis of the composition of each fiber individually, as all parameters that define fiber folding scale with the number of nucleosomes in the fiber.

**Table 2.** The fit results obtained from fitting multiple force-extension traces (mean ± SD)

	**Mono nucleosome**	15*197 NRL	30*167 NRL
*n*_fiber_	1	12 ± 4	27 ± 2
*k* (pN/nm)	-	0.22 ± 0.04	0.6 ± 0.2
*G*_1_(*k*_*B*_*T*)	8.8 ± 0.5	19 ± 2	18 ± 3
*G*_2_(*k*_*B*_*T*)	3.5 ± 1.0	4.4 ± 0.7	5.0 ± 0.4
Step size (nm)	24 ± 2	24 ± 7	24 ± 8
*n*_unfolded_	-	8 ± 6	10 ± 5

In addition to these parameters we fitted a constant offset. For the contourlength, persistence length and stretch modulus of the DNA, we used values obtained from the literature (see the Materials and Methods section).

### 167 NRL fibers are folded in a different manner than 197 NRL fibers

**Figure 5.**
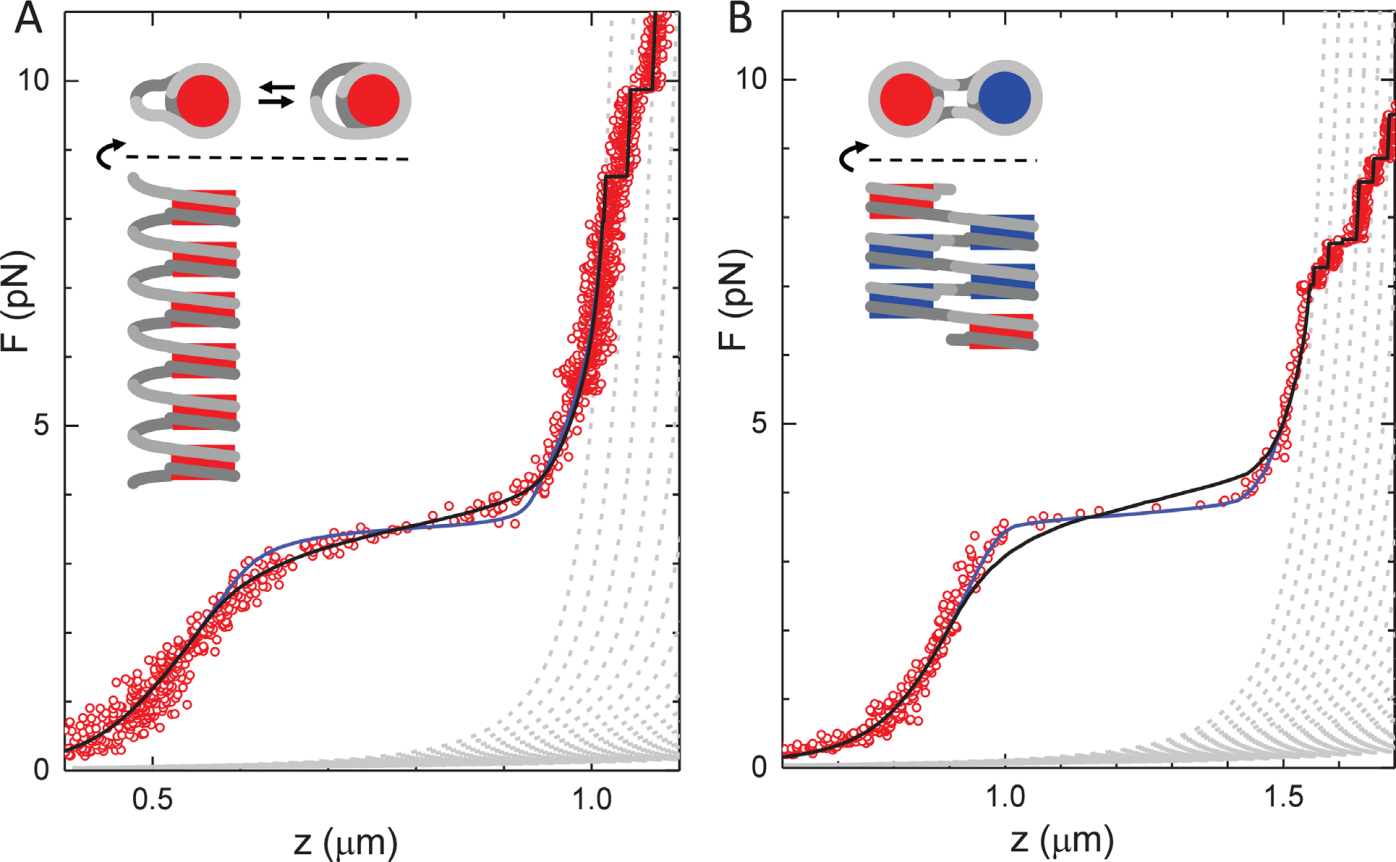
Chromatin fibers with 167-bp NRL follow a qualitatively different unfolding mechanism than 197-bp NRL fibers. (**A**) A 15*197 NRL chromatin fiber fits well with Equation (8), black line. A model in which the degeneracy for the first transition is lifted, blue line, does not capture the unfolding transitions. (**B**) A 30*167 NRL chromatin fiber is better described by non-degenerate states for the first transition. This qualitative difference can be explained by a different structure of the fibers, as tentatively sketched in the insets, showing both the top and the side views of the maximally extended fibers. In particular, the nucleosomes that are embedded in the fiber, drawn in blue in the schematic drawing of a zig-zag folded fiber, are less susceptible for unfolding than the red nucleosomes at the ends of the fiber. In contrast, the nucleosomes arranged in a single stack are all equivalent, inset of (A), and rupturing of any of the nucleosomes will lead to the same amount of extension of the fiber. The top view in (A) depicts possible unwrapping of nucleosomal DNA in the folded 197-bp NRL fiber.

The force-extension data of 197 NRL fibers closely follow the model based on independent transitions for all rupture events, including the first transition; see Figure 5A. This may be surprising in view of the large interaction energy and the high level of condensation up to 3 pN. Such independent rupturing can only be achieved when nucleosome–nucleosome interactions form exclusively between neighbors, as tentatively drawn in the inset of Figure 5A. Consistent with such a proposed structure, we note that the maximum extension per nucleosome, just before the first rupture event, is ∼13 nm, which can be spanned by the histone tails in a single file of stacked nucleosomes. Alternative organizations involving non-neighboring nucleosome–nucleosome interactions will generally result in more compact structures that cannot be stretched that much without rupturing those nucleosome–nucleosome contacts. Moreover, if non-neighboring nucleosomes would play a significant role in stabilizing chromatin folding, the nucleosomes at the ends would be more fragile than those embedded in the fiber. In fact, this scenario was already discussed by Cocco *et al*. (53), who argued that in that case, the degeneracy would be lifted for the transition. Indeed, removing the degeneracy in Equation (7) for the first transition does not give a good fit to the experimental curve, indicating that the data can best be interpreted in terms of interactions between neighboring nucleosomes only.

For chromatin arrays that have 20 bp of linker DNA the crystal structure of tetranucleosomes clearly shows stacking of non-neighboring nucleosomes (12). Cross-linking experiments further support a zig-zag folding in which odd and even nucleosomes interact into two parallel columns of nucleosomes (15). Such a structure would not only yield a two times smaller extension per nucleosome and a significantly higher stiffness, as we reported before (32), but also invalidate the independence of rupture events. Unlike the 197 NRL fibers, the force-extension curve of a 30*167 NRL chromatin fiber cannot be fitted with the degenerate unfolding model; see Figure 5B. The experimental data show a narrower force plateau as compared to the 197 NRL fiber. When the degeneracy of the first transition is taken out of the model (i.e. using *D*(state) = 1 in Equation (8)), a good fit is recovered. This not only changes the shape of the force-extension curve but also shifts the onset of the force plateau to a slightly higher value from 3.0 to 3.5 pN. As summarized in Table 2, all fit parameters, including the transition energy $\Delta G_{1}^{{\rm fiber}}$, are similar to those obtained for the 197 NRL fibers, except for the stiffness of the fiber. These observations reinforce the idea that 167 NRL and 197 NRL chromatins are arranged in a different structure.

## DISCUSSION

The folding of chromatin fibers and the mechanism of how they unfold under force have generated numerous debates. In this study we present and quantitatively interpret force spectroscopy on the unfolding of single chromatin fibers over a wide force range, spanning from less than 0.5 pN to more than 25 pN. These data include the well-studied high-force regime and allow for a detailed analysis of the entire stretching curve. The novelty of the introduced model lies in the treatment of each nucleosome of the fiber individually, as well as the direct coupling between the different force-dependent conformations of the nucleosomes in the force-extension curve. This approach strongly constraints the extension of possible conformations of the nucleosomes at various forces and properly takes into account the force-induced extension of both the bare DNA and the chromatin fiber.

Based on the measured extensions, we resolved a metastable conformation of the nucleosome, quantified the compositional heterogeneity of individual fibers in terms of number of nucleosomes and tetrasomes and showed that the unfolding mechanism of chromatin fibers is different for 197 NRL fibers that have been suggested to fold in a solenoid structure(13), as compared to 167 NRL fibers that fold in a zig-zag fashion (12). These data reinforce our previous structural interpretation of the force-extension curves (32) and allow for a detailed, quantitative comparison between fibers and with single nucleosomes, without biasing the results by selection of well-behaved fibers.

For a quantitative interpretation of the data it was essential to allow for compositional heterogeneity. This should not be surprising given the strong dependence of the reconstitution on the precise DNA/histone stoichiometry (13) and the known fragility of the nucleosome under typical single-molecule conditions (46). Moreover, we opted for a DNA construct with 1 kb of DNA on both sides of the chromatin fiber. Though such DNA extensions may allow for additional nucleosomes in the fiber, the long DNA handles proved useful to prevent or identify interactions between the reconstituted chromatin fiber and the surface of the flow cell or the bead. Though careful titration and handling of the sample can reduce this heterogeneity, we could not reliably produce perfectly defined fibers. Generally, we found that the number of nucleosomes that fold in a fiber reflects the number of Widom 601 positioning elements, but additional tetramers may be reconstituted and nucleosomes do partially dissociate into tetramers when exposed to excessive force over a longer time. This may be illustrative of the dynamics of chromatin *in vivo*, where H2A–H2B dimers are highly mobile (54,55). It also shows that assuming such perfect stoichiometry for single-molecule force spectroscopy may not be correct and that any analysis that does not take possible heterogeneity into account can be significantly flawed.

The novel extended conformation of the nucleosome between 3 and 7 pN that we report here explains the discrepancy between the reported stepsizes for the last unwrapping event, which vary between 20 and 30 nm, and the structural insight from the crystal structure, showing that a single wrap of DNA would constrain 89 bp, which would amount to 30 nm. The extension of the first unwrapped conformation is fully consistent with a single wrap nucleosome containing 89 bp of wrapped DNA, as explained previously by a spool model (40). However, for the last transition we measured a stepsize of 24 ± 7 nm. A metastable intermediate conformation, as sketched in Figure 2, can bridge the gap in extension between the first and the last rupture event. The necessity to include this conformation arises only when both force regimes are considered in the same experiment. Moreover, the additional extension is easily overlooked in the inherently noisy extension data, due to the high flexibility of a DNA tether at 3-pN force. In our pulling curves on chromatin fibers, the total extension scales with the number of nucleosomes, making it easier to resolve in chromatin arrays rather than single nucleosomes. Interestingly, in previous reports the larger stepsizes were typically observed in position clamps, whereas the smaller stepsizes, similar as reported here, were obtained in force clamps. The difference is in the pulling rates that can be rather high in position clamps, and could explain different kinetics along the unfolding pathway.

We can only speculate about the structural origin of the metastable extended conformation. It could be a nucleosome from which more DNA is unwrapped, a reorientation of the partially unwrapped nucleosome along the z-axis, a deformation of the remaining nucleosome core or (as tentatively sketched in Figure 2) the dissociation of H2A/H2B dimers from the histone core. It is clear though that any change in the orientation of the DNA exiting the nucleosome core can have a significant effect on the extension of the nucleosome, as reported here.

In our data we could not differentiate different classes of rupture events in the last transition, though all previous transitions only occur in nucleosomes that start off as fully folded. It is therefore likely that the novel metastable state structurally resembles that of a tetrasome under force. This interpretation could imply that the transition from a single wrap nucleosome to the extended conformation involves dissociation of the H2A–H2B dimers from histone core, rather than dissociation of DNA from the histone octamer. The transition is usually reversible, which is only possible when the H2A–H2B dimers remain bound to the DNA. Such a mechanism of nucleosome unfolding was recently resolved with single-molecule FRET in the absence of force (6). Preliminary force spectroscopy experiments in salt concentrations between 50 and 200 mM did not indicate a salt dependence of $z$_ext_ and Δ*G*_2_, but neither did the single-molecule FRET experiments in this range. Note that DNA does not extend from a tetrasome in exactly opposite directions, as it does in a single wrap nucleosome, which makes the force-extension relation non-trivial (38). Pending more detailed structural information of this conformation we therefore opt to model this conformation as having a constant extension in addition to a single wrap nucleosome. The forces at which these conformational changes take place are well within the range that may be expected *in vivo*, so this metastable conformation may have functional properties. Independent of its structure or function it is clear that this conformation should be included in a quantitative analysis of fiber unfolding under force.

We compared force-extension data of single nucleosomes with data of folded chromatin fibers with the same buffer conditions, histone composition and pulling protocol. As should be expected, single nucleosomes and nucleosomes embedded in chromatin fibers share the same stepwise unfolding pathway, except for the first transition into a single wrap nucleosome. This first transition involves a 10 *k*_*B*_*T* higher free energy per nucleosome in embedded nucleosomes than in a single nucleosome, which leads to a higher rupture force for DNA unwrapping from a chromatin fiber. Remarkably, the measured free energy of the folded conformation was the same for fibers with 197- and 167-bp NRL, despite possible different higher order structure of the fibers. The results that we obtained here with highly regular reconstituted chromatin fibers may therefore be more generic, and may be applicable for more disordered chromatin fibers, as found *in vivo*.

It is tempting to directly attribute the difference in free energy between the mononucleosome and a fiber embedded nucleosome to the nucleosome–nucleosome interaction energy. However, the situation may be more intricate. We could not resolve any indication of a fully wrapped nucleosome conformation in our fiber pulling data, i.e. a transition between the right two conformations drawn in Figure 2. This may simply be because the force for rupturing nucleosome–nucleosome interactions exceeds that of histone–DNA interactions, and when the nucleosomes are torn apart, DNA unwrapping directly follows within the time resolution of the experiment. Similar arguments however would apply to the transition into an extended nucleosome, which is clearly resolved as a broadening of the force plateau. Alternatively, it may be that the nucleosomes in the folded fiber are not fully wrapped and that part of the nucleosomal DNA is released from the histone core when the fiber folds into its higher order structure. FRET experiments on free nucleosomes have shown that unwrapping the first tens of bps of nucleosomal DNA is energetically not expensive (56). Such unwrapping would allow for less bending of the linker DNA, and may therefore be required for fiber folding. FRET experiments on nucleosomes in folded fibers may be able to test this hypothesis. Indirect evidence from restriction enzyme accessibility indicated that indeed nucleosomal DNA can be more accessible in chromatin fibers than in single nucleosomes (57), which do not have that constraint.

The comparable rupture energy Δ*G*^fiber^_1_ for 197 NRL and 167 NRL fibers may not be expected when the topology of the fibers is different. In fact, the anticipated difference in linker DNA bending energy between a solenoid and a zig-zag structure should reduce Δ*G*^fiber^_1_ for nucleosomes in a solenoidal structure. Partial unwrapping of nucleosomal DNA, as suggested above and tentatively depicted in the inset of Figure 5A, could however strongly relieve this tension. Alternative interpretations involving steric interactions between nucleosomes and/or zig-zag folding in 197 NRL fibers are difficult to reconcile with a linear extension and absence of hysteresis up to 3.5 pN. At this force we obtained an extension of 13 nm per nucleosome, which precludes many alternative conformations of the fiber.

Though the free energy difference is the same, the first rupture event is qualitatively different in fibers with different NRLs. It appears that nucleosomes in 197 NRL fibers rupture independently, whereas in 167 NRL fibers nucleosome rupture events appear to follow a cooperative mechanism. This observation is hard to reconcile with a gradual unwrapping of the first part of the wrapped DNA, as has been proposed before to explain the shape of the force-extension data (35), but quantitatively agrees with a different unfolding mechanism where nucleosomes are less stable at the ends of the fiber due to missing nucleosome–nucleosome interactions, as sketched in Figure 5B. In this scenario the nucleosomes would rupture sequentially from the ends, which is consistent with a solenoidal folding of 197 NRL fibers and a zig-zag folding of 167 NRL fibers. The maximum extension at the rupture force (13 versus 7 nm per nucleosome for 197- and 167-bp NRL fibers) and the almost four times higher stiffness for 167 NRL fibers also support this interpretation.

The very high reproducibility of the parameters that define the force-extension relation of chromatin fibers, both for the same fiber and between different fibers, suggests a well-defined folding mechanism. In principle, such a mechanism of fiber (un)folding would be accessible by structural modeling, rather than the simple empirical, Hookean model that we employ here. This would definitely provide more insight into the structure of the chromatin fiber. For single nucleosomes this has been successfully achieved using continuum models (27,38,40), and the data presented here largely agree with these results. For folded chromatin fibers however, there are many alternative compositions, structures and unfolding pathways. Though most aspects of the unfolding pathway presented here have been independently simulated in coarse-grained models, like the gradual unwrapping of DNA from nucleosome cores (35), the strict stacking of nucleosomes on each other (58), the dependence on the NRL (36) and extraction of nucleosome interaction energies from force-extension curves (59), the current data and quantitative description may help to refine such structural models, which are generally not compatible at the current state.

Despite the complexity of the fiber we were able to resolve a clear mechanism of fiber unfolding that is consistent for various architectures of chromatin. With the model and the parameters that described force-induced structural changes in chromatin it should now be possible to resolve the effects of post-translational modifications on the structure and dynamics of chromatin at the molecular scale. It should also be possible to extend the experiments and model to torsionally constraint topological domains of chromatin. In addition, because we can describe the mechanics of chromatin fibers at the level of individual nucleosomes, it will be interesting to move toward fibers that are heterogeneous in terms of linker length, mimicking the situation *in vivo* more closely. These steps will lead to a fundamental structural understanding of chromatin fiber folding, without oversimplification or imposing regularity that is often required to interpret structural data.
